# TCGA and ESTIMATE data mining to identify potential prognostic biomarkers in HCC patients

**DOI:** 10.18632/aging.103943

**Published:** 2020-11-11

**Authors:** Guolin He, Shunjun Fu, Yang Li, Ting Li, Purong Mei, Lei Feng, Lei Cai, Yuan Cheng, Chenjie Zhou, Yujun Tang, Wenbin Huang, Haiyan Liu, Bohong Cen, Mingxin Pan, Yi Gao

**Affiliations:** 1 Department of Hepatobiliary Surgery, Zhujiang Hospital, Southern Medical University, Guangzhou 510282, China; 2 Department of Pharmacy, Zhujiang Hospital of Southern Medical University, Guangzhou 510282, Guangdong, China; 3 Department of Radiation Oncology, Affiliated Cancer Hospital and Institute of Guangzhou Medical University, Guangzhou 510095, Guangdong, China

**Keywords:** TCGA, GEO, tumor microenvironment, immune scores, disease-free survival

## Abstract

Hepatocellular carcinoma (HCC) is an aggressive form of cancer characterized by a high recurrence rate following resection. Studies have implicated stromal and immune cells, which form part of the tumor microenvironment, as significant contributors to the poor prognoses of HCC patients. In the present study, we first downloaded gene expression datasets for HCC patients from The Cancer Genome Atlas database and categorized the patients into low and high stromal or immune score groups. By comparing those groups, we identified differentially expressed genes significantly associated with HCC prognosis. The Gene Ontology database was then used to perform functional enrichment analysis, and the STRING network database was used to construct protein-protein interaction networks. Our results show that most of the differentially expressed genes were involved in immune processes and responses and the plasma membrane. Those results were then validated using another a dataset from a HCC cohort in the Gene Expression Omnibus database and in 10 pairs of HCC tumor tissue and adjacent nontumor tissue. These findings enabled us to identify several tumor microenvironment-related genes that associate with HCC prognosis, and some those appear to have the potential to serve as HCC biomarkers.

## INTRODUCTION

Hepatocellular carcinoma (HCC) is a prevalent malignant tumor and a leading cause of cancer-associated death globally [1]. More than 50% of the world's HCC occurs in China, and the incidence rate has been increased yearly [2]. The 5-year survival rate among advanced HCC patients is less than 5% due to the disease’s high recurrence and metastasis rates [3]. The occurrence and development of HCC is a multifactorial, multistage process that involves the hepatocytes themselves as well as their microenvironment [4].

Previous studies have shown that the tumor microenvironment not only influences gene expression in HCC, but also the clinical outcomes of the patients [5–7]. Immune and stromal cells are vital nontumor components of the tumor microenvironment and can be used for diagnostic and prognostic evaluation. The immune score is a standard method for quantifying T cell and cytotoxic T cell density within a tumor microenvironment and provides data that are predictive of patient outcomes [8]. Immune and matrix scores are calculated using the ESTIMATE (Estimation of STromal and Immune cells in MAlignant Tumor tissues using Expression data) algorithm. This method facilitates quantification of immune and matrix constituents within tumors [9], and there is increasing evidence that an inflammatory environment is predictive of clinical outcome [10–13].

To better understand the impact of stromal and immune cell-related genes on prognosis, in the present study we systematically analyzed tumor expression profiles and explored tumor microenvironment-related genes associated with a poor prognosis and their potential regulatory mechanisms. We initially analyzed HCC cohorts in The Cancer Genome Atlas database and used ESTIMATE immune scores to predicted genes that significantly affect outcomes in HCC patients. We then used the string database to perform functional annotation of these genes, after which we used qRT-PCR to verify expression of eight genes of interest in clinical samples and TCGA database. Finally, we performed a survival analysis to verify the impact of the eight genes of interest using a different HCC dataset from the Gene Expression Omnibus database (GSE14520).

## RESULTS

### Immune scores are significantly related to disease-free survival in HCC

**Figure 1:**
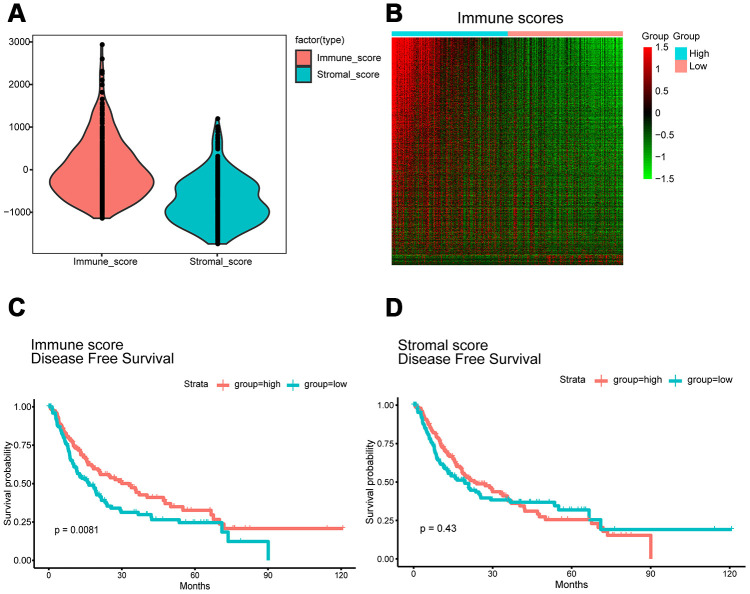
**Immune scores and stromal scores are associated with HCC disease-free survival.** (**A**) TCGA liver cancer expression profile data using ESTIMATE method to calculate immune score and matrix score. Box-plot shows that the level of Immune scores and stromal scores. (**B**) Heatmap of the DEGs of immune scores of top half (high score) vs. bottom half (low score). p<0.05, fold change >1). Genes with higher expression are shown in red, lower expression are shown in green, genes with same expression level are in black. (**C**) HCC cases were divided into two groups based on their immune scores. Median disease-free survival of the high score group is longer than low score group (log-rank test, p<0.05). (**D**) Similarly, HCC cases were divided into two groups based on their stromal scores. The median disease-free survival of the low score group is longer than the high score group (log-rank test p=0.43), however, it is not statistically different.

Gene expression profiles with pathologic diagnosis and clinical data from 319 HCC patients were retrieved from TCGA database. Based on the ESTIMATE algorithm, stromal scores ranged from -1,741.56 to 1,195.07, and immune scores ranged from -1,209.16 to 2,934.36 (Figure 1A). The patients were then divided into high and low score (divided based on median score) groups to determine the potential correlation between disease-free survival and immune or stromal scores. Kaplan-Meier survival curves revealed that patients in the high immune score group had a longer median disease-free survival time than those in the low score group (p = 0.0081 in log-rank test). (Figure 1C and 1D).

### Correlation between gene expression and immune scores

We next evaluated the 319 HCC cases from TCGA database to assess the relationship between gene expression and immune scores. Using a fold change of |logFC |> 1 and significance threshold of P <0.05, we selected 1195 differentially expressed genes (DEGs), of which 1144 were overexpressed and 51 were underexpressed. A heat map of the DEGs is shown in Figure 1B. Each row represents one gene, and each column represents one sample. The samples are sorted from left to right based on the immune score; the blue group on the left are the samples from the high score group, while the pink group on the right are the samples from the low score group. The genes were ranked according to the P-value of the differential expression analysis from smaller to larger. Red indicates overexpression, while green indicates underexpression, and the darker the red or green color, the greater the difference in expression.

**Figure 2:**
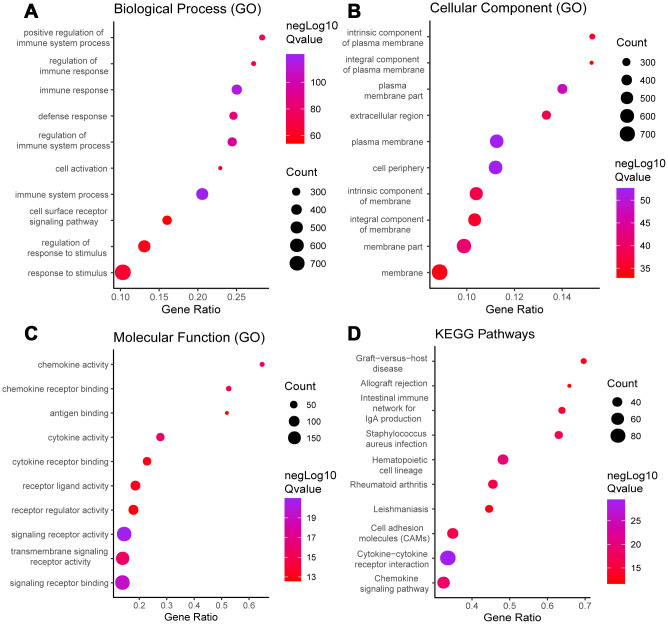
**GO term and KEGG pathway analysis for all DEGs.** Top 10 GO terms. False discovery rate (FDR) of GO analysis was acquired from STRING database. p <0.05. (**A**) biological process, (**B**) cellular component, (**C**) molecular function, and (**D**) KEGG pathway.

Functional enrichment analysis was performed to study the function of the DEGs. Gene Ontology (GO) analysis revealed that the DEGs were involved with “plasma membrane,” “immune process,” “immune response,” and “signaling receptor” activities. In addition, Kyoto Encyclopedia of Genes and Genomes (KEGG) pathway analysis showed significant involvement of the chemokine signaling pathway and cytokine-cytokine receptor interaction pathway. Figure 2 shows the top ten enrichment results for GO annotation and KEGG pathway enrichment, and Supplementary Tables 1–4 list the specific enrichment information.

**Figure 3:**
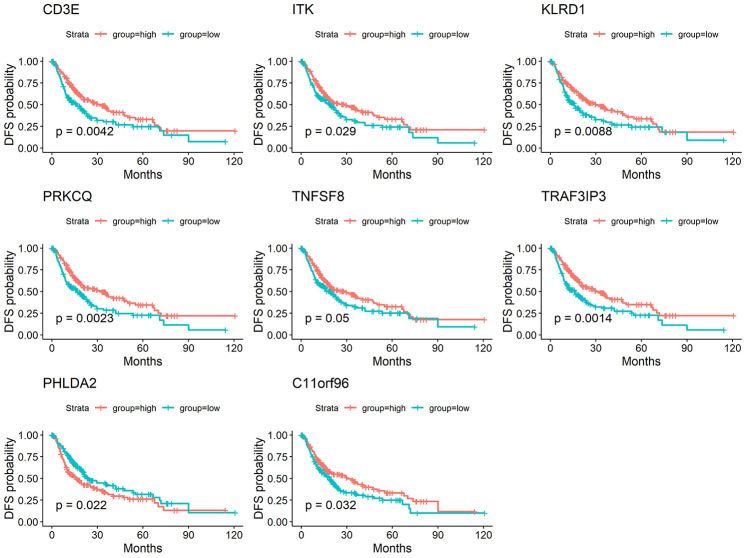
**Correlation of expression of individual DEGs in disease-free survival in TCGA.** Kaplan-Meier survival curves were generated for selected DEGs extracted from the comparison of groups of high (red line) and low (blue line) gene expression. p<0.05 in Log-rank test. DFS, disease-free survival.

We performed a survival analysis and generated Kaplan-Meier survival plots to examine the possible impact of each DEG on disease-free survival. Of the 1195 DEGs, 214 predicted poor or good disease-free survival in the log-rank test (Supplementary File 1 list the total 214 genes, Figure 3 list 8 of 214 DEGs) (p<0.05).

### Protein-protein interactions network and functional enrichment analysis from genes of significant value

**Figure 4:**
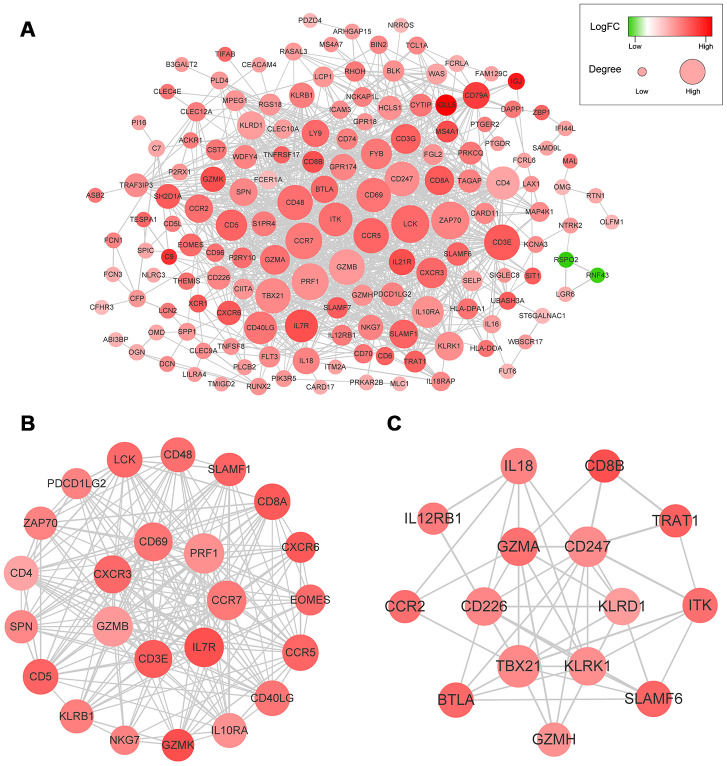
(**A**) The whole PPI networks of the 214 predictive DEGs. (**B**) The module 1 of the two most significant modules in the whole PPI network. (**C**) The module 2 of the two most significant modules in the whole PPI network.

We used the STRING database to generate protein-protein interaction (PPI) networks to explore the interactions among the 214 predictive DEGs. We detected 7 modules in a network that included 156 nodes and 1,049 edges (Figure 4A). The two most significant modules were selected for subsequent analysis. In module 1 (Figure 4B), 199 edges involving 24 nodes were formed within the network. The genes, with the most connections to the immune response included *PRF1, CCR7, IL7R, CD3E, GZMB, CXCR3, CCR5, CD4, CD40LG, CD8A, EOMES, LCK, PDCD1LG2, SLAMF1, SPN, ZAP70, CD48* and *CD5*. In module 2 (Figure 4C), critical genes related to immune responses, including *GZMA, KLRK1, KLRD1, IBTLA, CCR2, CD8B, L12RB1, IL18, ITK, SLAMF6* and *TRAT1*, were located at the center of the module.

**Figure 5:**
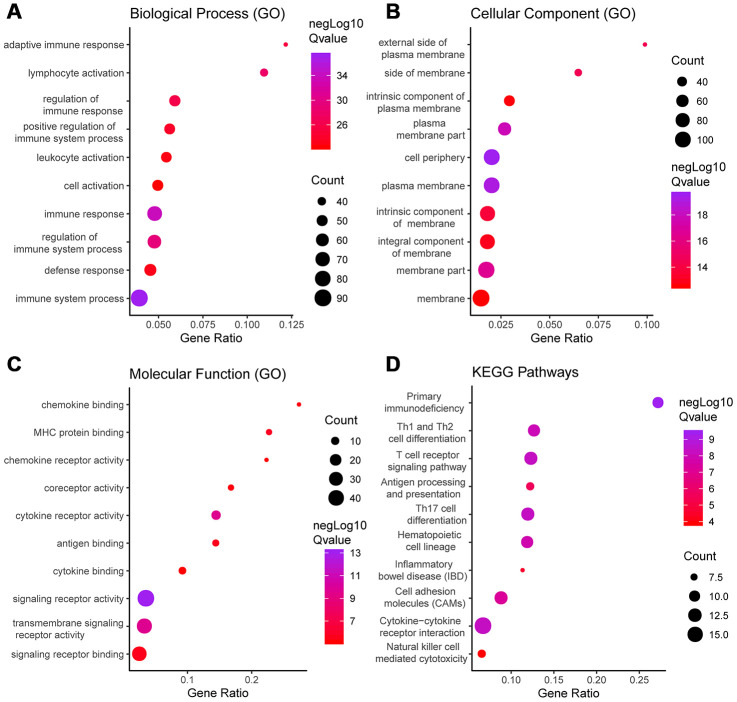
**GO term and KEGG pathway analysis for DEGs significantly associated with disease-free survival.** Top pathways with FDR < 0.05, -log FDR >1.301 are shown: (**A**) biological process, (**B**) cellular component, (**C**) molecular function, and (**D**) KEGG pathway.

Functional enrichment clustering of these genes exhibited a strong association with immune responses. Top 10 GO terms included “signaling receptor activity,” “immune response,” “immune system process,” and “cell periphery, extracellular region.” In addition, KEGG pathway analysis showed all the pathways were associated with immune responses (Figure 5A–5D). Significant GO terms identified include 5 for “molecular function,” 12 for “cellular component” and 30 for “biological process.” Supplementary Tables 5–8 lists the specific enrichment information.

### GEO database and clinical sample validation

**Figure 6:**
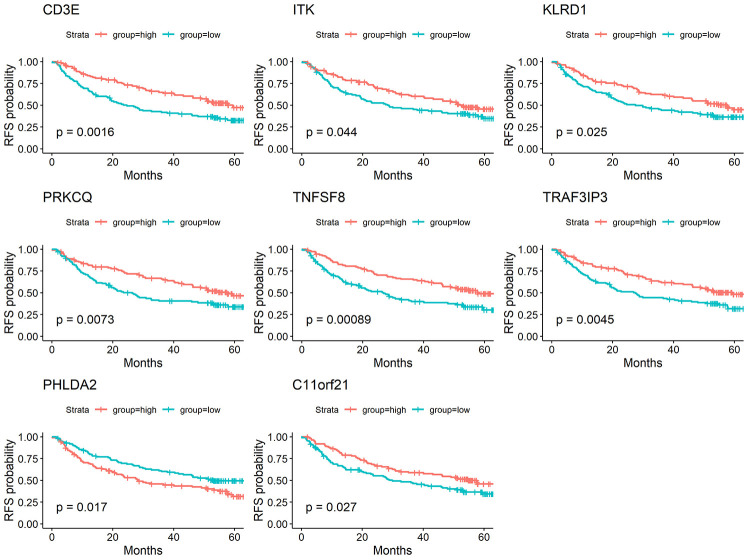
**Validation of DEGs extracted from TCGA database with disease-free survival in GEO cohort.** Kaplan-Meier survival curves were generated for selected DEGs extracted from the comparison of groups of high (red line) and low (blue line) gene expression. p<0.05 in Log-rank test. DFS, disease-free survival.

**Figure 7:**
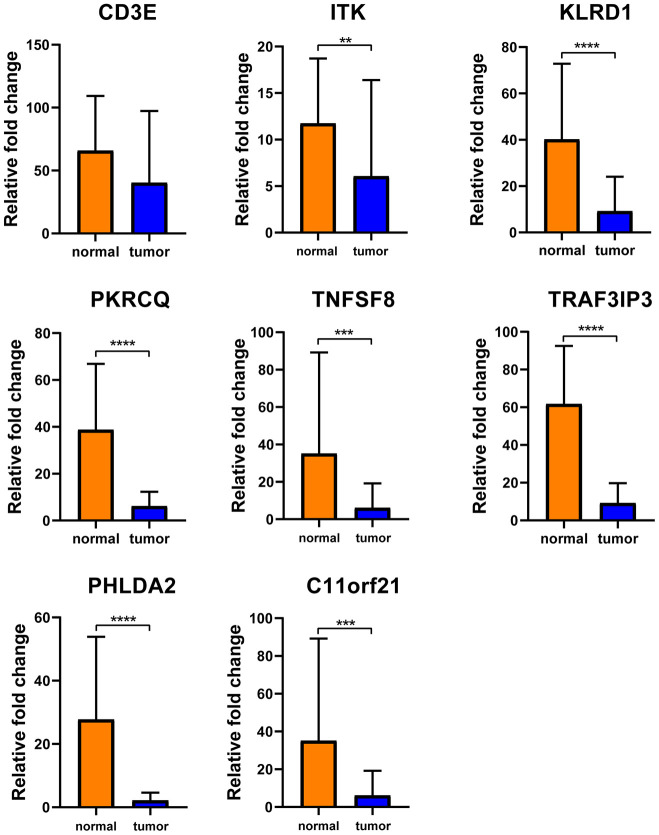
**Verification of these 8 interested DEGs in clinical samples.** Relative mRNA levels of 8 genes in 10 HCC samples were frequently overexpressed in nontumor tissues compared with matched HCC tissues(p<0.05) by qRT-PCR except CD3E.

To determine whether the aforementioned genes are also of prognostic significance in other HCC cases, a gene expression dataset (GSE14520) from a different HCC cohort (221 cases) were downloaded and analyzed. Among the 214 predictive DEGs, 13 were confirmed to be significantly associated with clinical prognosis. Of those, eight genes (*TNFSF8, CD3E, ITK, KLRD1, PRKCQ, TRAF3IP3, PHLDA2, C11orf21*) were of particular interest because their differential expression had not been previously reported in HCC patients (Figure 6). We used qRT-PCR to assess expression of these eight genes of interest in 10 pairs of fresh HCC and adjacent nontumor tissues. The results showed that levels of the transcripts of these eight genes were frequently higher (p<0.05) in the corresponding nontumor tissues than to the HCC tissues (Figure 7).

### Correlation between expression of genes of interested and expression of immune checkpoint gene

**Figure 8:**
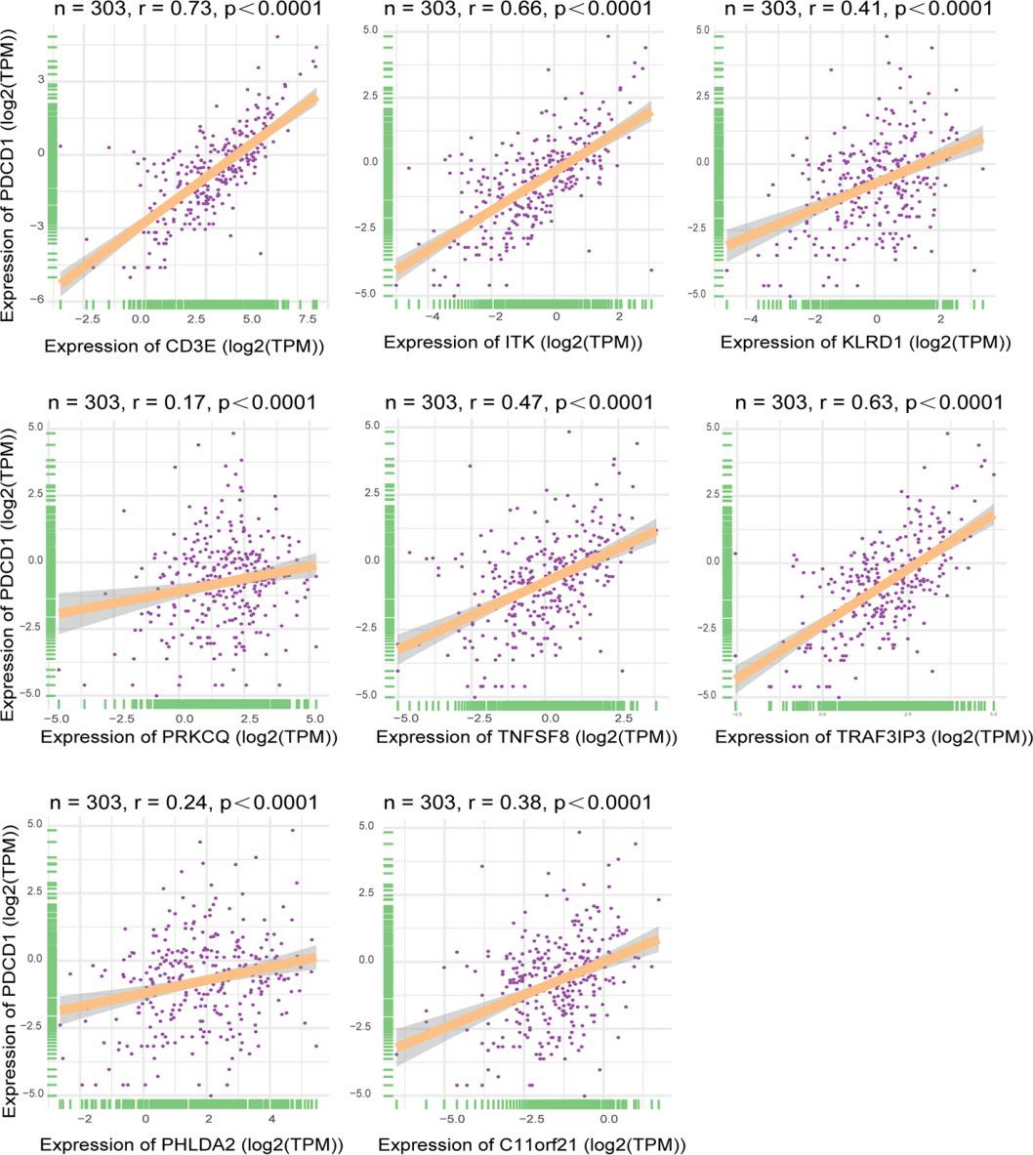
**Correlation between expression of interested DEGs and immune checkpoint gene.** Pearson correlation of expression and ImmuneScore dataset. The all 8 interested genes had significant correlation(p<0.05), especially CD3E, ITK and TRAF3IP3. X-axis represented expression level of 8 interested genes in each sample. Y-axis represented expression level of PDCD1 in each sample.

Using TCGA datasets, we found that expression of the eight genes of interest correlated positively with the mRNA expression of the immune checkpoint gene *PDCD1* (Figure 8) (p<0.05). Of the eight genes, the strongest correlation was between *CD3E* and *PDCD1*.

## DISCUSSION

In the present study, we used a dataset from TCGA to identify genes related to the tumor microenvironment that influenced disease-free survival. By comparing between groups with high or low immune scores and performing a GO term functional analysis, we initially extracted 1195 differentially expressed genes involved in the tumor microenvironment. We then carried out a disease-free survival analysis, which revealed that 214 of the genes were related to poor disease outcomes in HCC patients. Finally, using a validation cohort from the GEO database (GSE14520), we detected 13 genes related to tumor microenvironment that correlated significantly with prognosis. Of those five (*GZMA, CD79A, IGJ, CYP3A4, SPP1*) are reportedly predictive of overall survival or are involved in HCC pathogenesis (Supplementary Table 9). The remaining eight genes, including *TNFSF8, CD3E, ITK, KLRD1, PRKCQ, TRAF3IP3, PHLDA2*, and *C11orf21*, have not previously been reported to impact the clinical prognosis of HCC patients, and may have the potential to serve as new biomarkers for HCC. There was also a strong relationship between these eight genes, especially *CD3E, ITK* and *TRAF3IP3*, and the immune checkpoint gene *PDCD1*, which suggests HCC patients showing high expression of CD3E, ITK and TRAF3IP3 may respond well to immunotherapy.

PPI network analysis revealed interleukin-7 receptor (IL7R) and killer cell lectin-like receptor K1 (KLRK1) to be highly interconnected nodes (Figure 4). IL7R plays a vital role in lymphocyte development. For example, IL7R-deficiency may contribute to severe combined immunodeficiency (SCID). Moreover, it was recently reported that IL7R is associated with the risk of HCC [14–16]. KLRK1, also called NKG2D (NKG2-D-activating NK receptor), binds noncovalently with the DAP10 signaling protein to deliver costimulatory or activating signals to T and NK cells. Sheppard et al. suggested NKG2D promotes tumor growth in a chronic inflammation model of HCC [17, 18].

Statistics from several large-scale clinical trials of cancer patients demonstrate that the number, type, and region of infiltrating lymphocytes within tumor tissue are decisive for predicting clinical outcomes [8, 19–21]. Previous studies of colon cancer [22], ovarian cancer [23, 24], lung cancer [25], and melanoma [26] showed that immune cell activation-related genes are upregulated in patients with good prognoses. Pages et al. [27] used immunological scoring methods to follow up the survival and recurrence in stage I and II colon cancer patients. They found that both overall and disease-free survival were significantly better in patients with higher immune scores than in those with lower immune scores. Patients with an immune score of 4 had more prolonged overall survival, and 95% of these patients had no tumor recurrence within 18 years after surgery. On the other hand, 50% of patients with an immune score of 0 had tumor recurrence within 2 years after surgery. In the present study, higher immune scores associated with better prognosis in HCC patients, which is consistent with that earlier report. These findings may offer a different perspective on the complex interaction between tumors and their immune environment in HCC. However, there remains a need for further studies on these genes, which we anticipate will provide additional new insight into the impact of the tumor microenvironment in HCC.

## MATERIALS AND METHODS

### Source of data

Gene expression profiles, as well as patients' clinical information, were obtained from TCGA database (https://tcga-data.nci.nih.gov/tcga/). Immune and stromal scores were calculated using the ESTIMATE algorithm (https://bioinformatics.mdanderson.org/estimate/). The validation dataset (GSE14520) was extracted from the GEO database (https://www.ncbi.nlm.nih.gov/geo/)

### ESTIMATE algorithm

ESTIMATE (Estimation of STromal and Immune cells in MAlignant Tumour tissues using Expression data) is an algorithmic tool. The detail algorithm can be seen at Supplementary File 3. Yoshihara and his colleagues [28] calculate stromal and immune scores to predict the level of infiltrating stromal and immune cells through single-sample gene set-enrichment analysis (ssGSEA), and these scores make up the basis of the ESTIMATE score to infer tumour purity in tumour tissue.

### Clinical samples

Ten pairs of fresh HCC specimens and adjacent nontumor tissue were collected from Southern Medical University, Zhujiang Hospital (Guangzhou, China). The human specimens used for validation in this study were collected between May and June 2020. Use of these specimens was approved by the local ethics committee. The adjacent samples were taken at a distance of at least 5 cm from the tumor, and all tissues were examined histologically. None of the patients had received preoperative chemotherapy or radiotherapy.

### RNA extraction and quantitative real-time polymerase chain reaction(qRT-PCR)

Total RNA was extracted from the tissue specimens using TRIzol (Invitrogen), and qRT-PCR was performed with SYBR Green Dye (Takara, Dalian, China) according to the manufacturer’s instructions. The primer sequences are listed in Supplementary File 2.

### Identification of DEGs and Heatmap and clustering analysis

Data were analyzed using R software (version 3.6.2) and its package Limma. |logFC|>1 and p < 0.05 were set as the cut-offs to screen for DEGs. Heatmaps and clustering were generated using the R software.

### PPI network construction

The STRING network database (https://string-db.org/) was employed for construction of PPI (protein-protein interaction) networks using the Cytoscape app. We then selected specific networks with ten or more nodes for subsequent analysis, and the degree of connectivity for each network node was calculated. To detect densely connected regions, we used the Cytoscape plug-in clustering algorithm called Molecular COmplex DEtection (MCODE) to identify clusters based on their topological features.

### Recurrence-free survival curve analysis

We carried out a Kaplan-Meier analysis using survival R package to assess the association between recurrence-free survival among patients and the levels of DEG expression. The constructed curves were compared using the log-rank test.

### Enrichment analysis of DEGs

We used the STRING network database to perform functional enrichment and pathway enrichment analyses of the DEGs. The enrichment content included GO, Reactome Pathways, Kyoto Encyclopedia of Genes and Genomes (KEGG) Pathways, UniProt Keywords, PFAM Protein Domains, SMART Protein Domains, INTERPRO Protein Domains and Features, and other databases. The pathway enrichment analysis was based on references from the KEGG pathways. A false discovery rate (FDR) < 0.05 was used as the cut-off.

### Correlation analysis

To explore the relationship of the 8 novel genes identified through the data mining with immune checkpoint gene (PDCD1) expression, We used TCGA database and R software to perform correlation analyses.

## Supplementary Material

Supplementary Tables

Supplementary File 1

Supplementary File 2

Supplementary File 3

## References

[1] Waly Raphael S, Yangde Z, Yuxiang C. Hepatocellular carcinoma: focus on different aspects of management. ISRN Oncol. 2012; 2012:421673. 10.5402/2012/421673 22655206 PMC3359687

[2] McGlynn KA, London WT. The global epidemiology of hepatocellular carcinoma: present and future. Clin Liver Dis. 2011; 15:223–43. 10.1016/j.cld.2011.03.006 21689610 PMC4141529

[3] Lee JG, Kang CM, Park JS, Kim KS, Yoon DS, Choi JS, Lee WJ, Kim BR. The actual five-year survival rate of hepatocellular carcinoma patients after curative resection. Yonsei Med J. 2006; 47:105–12. 10.3349/ymj.2006.47.1.105 16502491 PMC2687566

[4] Hernandez-Gea V, Toffanin S, Friedman SL, Llovet JM. Role of the microenvironment in the pathogenesis and treatment of hepatocellular carcinoma. Gastroenterology. 2013; 144:512–27. 10.1053/j.gastro.2013.01.002 23313965 PMC3578068

[5] Wu T, Dai Y. Tumor microenvironment and therapeutic response. Cancer Lett. 2017; 387:61–68. 10.1016/j.canlet.2016.01.043 26845449

[6] Azizi E, Carr AJ, Plitas G, Cornish AE, Konopacki C, Prabhakaran S, Nainys J, Wu K, Kiseliovas V, Setty M, Choi K, Fromme RM, Dao P, et al. Single-cell map of diverse immune phenotypes in the breast tumor microenvironment. Cell. 2018; 174:1293–308.e36. 10.1016/j.cell.2018.05.060 29961579 PMC6348010

[7] Pearce OM, Delaine-Smith RM, Maniati E, Nichols S, Wang J, Böhm S, Rajeeve V, Ullah D, Chakravarty P, Jones RR, Montfort A, Dowe T, Gribben J, et al. Deconstruction of a metastatic tumor microenvironment reveals a common matrix response in human cancers. Cancer Discov. 2018; 8:304–19. 10.1158/2159-8290.CD-17-0284 29196464 PMC5837004

[8] Hendry S, Salgado R, Gevaert T, Russell PA, John T, Thapa B, Christie M, van de Vijver K, Estrada MV, Gonzalez-Ericsson PI, Sanders M, Solomon B, Solinas C, et al. Assessing Tumor-infiltrating Lymphocytes in Solid Tumors: A Practical Review for Pathologists and Proposal for a Standardized Method From the International Immunooncology Biomarkers Working Group: Part 1: Assessing the Host Immune Response, TILs in Invasive Breast Carcinoma and Ductal Carcinoma In Situ, Metastatic Tumor Deposits and Areas for Further Research. Adv Anat Pathol. 2017; 24:235–251. 10.1097/PAP.0000000000000162 28777142 PMC5564448

[9] Pan XB, Lu Y, Huang JL, Long Y, Yao DS. Prognostic genes in the tumor microenvironment in cervical squamous cell carcinoma. Aging (Albany NY). 2019; 11:10154–10166. 10.18632/aging.102429 31740624 PMC6914434

[10] Alonso MH, Aussó S, Lopez-Doriga A, Cordero D, Guinó E, Solé X, Barenys M, de Oca J, Capella G, Salazar R, Sanz-Pamplona R, Moreno V. Comprehensive analysis of copy number aberrations in microsatellite stable colon cancer in view of stromal component. Br J Cancer. 2017; 117:421–31. 10.1038/bjc.2017.208 28683472 PMC5537504

[11] Priedigkeit N, Watters RJ, Lucas PC, Basudan A, Bhargava R, Horne W, Kolls JK, Fang Z, Rosenzweig MQ, Brufsky AM, Weiss KR, Oesterreich S, Lee AV. Exome-capture RNA sequencing of decade-old breast cancers and matched decalcified bone metastases. JCI Insight. 2017; 2:e95703. 10.1172/jci.insight.95703 28878133 PMC5621874

[12] Shah N, Wang P, Wongvipat J, Karthaus WR, Abida W, Armenia J, Rockowitz S, Drier Y, Bernstein BE, Long HW, Freedman ML, Arora VK, Zheng D, Sawyers CL. Regulation of the glucocorticoid receptor via a BET-dependent enhancer drives antiandrogen resistance in prostate cancer. Elife. 2017; 6:e27861. 10.7554/eLife.27861 28891793 PMC5593504

[13] Jia D, Li S, Li D, Xue H, Yang D, Liu Y. Mining TCGA database for genes of prognostic value in glioblastoma microenvironment. Aging (Albany NY). 2018; 10:592–605. 10.18632/aging.101415 29676997 PMC5940130

[14] Kong F, Hu W, Zhou K, Wei X, Kou Y, You H, Zheng K, Tang R. Hepatitis B virus X protein promotes interleukin-7 receptor expression via NF-κB and Notch1 pathway to facilitate proliferation and migration of hepatitis B virus-related hepatoma cells. J Exp Clin Cancer Res. 2016; 35:172. 10.1186/s13046-016-0448-2 27821177 PMC5100324

[15] Kondo Y, Ueno Y, Kobayashi K, Kakazu E, Shiina M, Inoue J, Tamai K, Wakui Y, Tanaka Y, Ninomiya M, Obara N, Fukushima K, Ishii M, et al. Hepatitis B virus replication could enhance regulatory T cell activity by producing soluble heat shock protein 60 from hepatocytes. J Infect Dis. 2010; 202:202–13. 10.1086/653496 20533879

[16] Li L, Guo L, Wang Q, Liu X, Zeng Y, Wen Q, Zhang S, Kwok HF, Lin Y, Liu J. DAPK1 as an independent prognostic marker in liver cancer. PeerJ. 2017; 5:e3568. 10.7717/peerj.3568 28740751 PMC5520959

[17] Sheppard S, Guedes J, Mroz A, Zavitsanou AM, Kudo H, Rothery SM, Angelopoulos P, Goldin R, Guerra N. The immunoreceptor NKG2D promotes tumour growth in a model of hepatocellular carcinoma. Nat Commun. 2017; 8:13930. 10.1038/ncomms13930 28128200 PMC5290164

[18] Sheppard S, Ferry A, Guedes J, Guerra N. The paradoxical role of NKG2D in cancer immunity. Front Immunol. 2018; 9:1808. 10.3389/fimmu.2018.01808 30150983 PMC6099450

[19] Rohaan MW, van den Berg JH, Kvistborg P, Haanen JB. Adoptive transfer of tumor-infiltrating lymphocytes in melanoma: a viable treatment option. J Immunother Cancer. 2018; 6:102. 10.1186/s40425-018-0391-1 30285902 PMC6171186

[20] Nguyen LT, Saibil SD, Sotov V, Le MX, Khoja L, Ghazarian D, Bonilla L, Majeed H, Hogg D, Joshua AM, Crump M, Franke N, Spreafico A, et al. Phase II clinical trial of adoptive cell therapy for patients with metastatic melanoma with autologous tumor-infiltrating lymphocytes and low-dose interleukin-2. Cancer Immunol Immunother. 2019; 68:773–85. 10.1007/s00262-019-02307-x 30747243 PMC11028227

[21] Perez EA, Ballman KV, Tenner KS, Thompson EA, Badve SS, Bailey H, Baehner FL. Association of stromal tumor-infiltrating lymphocytes with recurrence-free survival in the N9831 adjuvant trial in patients with early-stage HER2-positive breast cancer. JAMA Oncol. 2016; 2:56–64. 10.1001/jamaoncol.2015.3239 26469139 PMC4713247

[22] Fehlker M, Huska MR, Jöns T, Andrade-Navarro MA, Kemmner W. Concerted down-regulation of immune-system related genes predicts metastasis in colorectal carcinoma. BMC Cancer. 2014; 14:64. 10.1186/1471-2407-14-64 24495478 PMC3922093

[23] Drakes ML, Stiff PJ. Regulation of ovarian cancer prognosis by immune cells in the tumor microenvironment. Cancers (Basel). 2018; 10:302. 10.3390/cancers10090302 30200478 PMC6162424

[24] Menyhárt O, Fekete JT, Győrffy B. Gene expression indicates altered immune modulation and signaling pathway activation in ovarian cancer patients resistant to topotecan. Int J Mol Sci. 2019; 20:2750. 10.3390/ijms20112750 31195594 PMC6600443

[25] Carbone DP, Gandara DR, Antonia SJ, Zielinski C, Paz-Ares L. Non-small-cell lung cancer: role of the immune system and potential for immunotherapy. J Thorac Oncol. 2015; 10:974–84. 10.1097/JTO.0000000000000551 26134219 PMC4618296

[26] Passarelli A, Mannavola F, Stucci LS, Tucci M, Silvestris F. Immune system and melanoma biology: a balance between immunosurveillance and immune escape. Oncotarget. 2017; 8:106132–42. 10.18632/oncotarget.22190 29285320 PMC5739707

[27] Pagès F, Mlecnik B, Marliot F, Bindea G, Ou FS, Bifulco C, Lugli A, Zlobec I, Rau TT, Berger MD, Nagtegaal ID, Vink-Börger E, Hartmann A, et al. International validation of the consensus immunoscore for the classification of colon cancer: a prognostic and accuracy study. Lancet. 2018; 391:2128–39. 10.1016/S0140-6736(18)30789-X 29754777

[28] Yoshihara K, Shahmoradgoli M, Martínez E, Vegesna R, Kim H, Torres-Garcia W, Treviño V, Shen H, Laird PW, Levine DA, Carter SL, Getz G, Stemke-Hale K, et al. Inferring tumour purity and stromal and immune cell admixture from expression data. Nat Commun. 2013; 4:2612. 10.1038/ncomms3612 24113773 PMC3826632

