# Supplementary material for: TCGA and ESTIMATE data mining to identify potential prognostic biomarkers in HCC patients
Source: Aging (Albany NY). 2020 Nov 11;12(21):21544–58. doi: 10.18632/aging.103943 (PMC7695391; doi:10.18632/aging.103943)
Supplement: Supplementary Tables [file aging-12-103943-s001..pdf]

SUPPLEMENTARY TABLES

Supplementary Table 1. Top 10 GO terms enrichment analysis in the category molecular function of differentially expressed genes.

Term ID	Description	Observed gene count	Background gene count	FDR
GO:0038023	signaling receptor activity	197	1429	1.58E-21
GO:0005102	signaling receptor binding	199	1513	4.68E-20
GO:0004888	transmembrane signaling receptor activity	163	1226	1.91E-16
GO:0005125	cytokine activity	59	216	1.91E-16
GO:0008009	chemokine activity	31	48	3.05E-16
GO:0042379	chemokine receptor binding	33	63	2.34E-15
GO:0048018	receptor ligand activity	83	458	3.12E-14
GO:0005126	cytokine receptor binding	61	272	7.99E-14
GO:0030545	receptor regulator activity	84	483	1.33E-13
GO:0003823	antigen binding	29	56	1.73E-13

Supplementary Table2. Top 10 GO terms enrichment analysis in the category biological process of differentially expressed genes.

Term ID	Description	Observed gene count	Background gene count	FDR
GO:0002376	immune system process	483	2370	3.92E-120
GO:0006955	immune response	388	1560	3.24E-115
GO:0002682	regulation of immune system process	338	1391	1.35E-95
GO:0006952	defense response	302	1234	1.24E-84
GO:0002684	positive regulation of immune system process	249	882	8.15E-79
GO:0050776	regulation of immune response	237	873	7.77E-72
GO:0050896	response to stimulus	785	7824	9.92E-65
GO:0048583	regulation of response to stimulus	500	3882	6.17E-60
GO:0001775	cell activation	234	1024	6.76E-59
GO:0007166	cell surface receptor signaling pathway	349	2198	2.74E-56

Supplementary Table 3. Top 10 GO terms enrichment analysis in the category cellular component of differentially expressed genes.

Term ID	Description	Observed gene count	Background gene count	FDR
GO:0071944	cell periphery	585	5254	6.64E-53
GO:0005886	plasma membrane	577	5159	9.9E-53
GO:0044459	plasma membrane part	370	2651	3.34E-48
GO:0044425	membrane part	639	6517	1.35E-41
GO:0031224	intrinsic component of membrane	549	5316	5.93E-39
GO:0005576	extracellular region	333	2505	1.51E-38
GO:0016021	integral component of membrane	529	5155	3.11E-36
GO:0031226	intrinsic component of plasma membrane	250	1641	4.94E-36
GO:0016020	membrane	739	8420	4.57E-35
GO:0005887	integral component of plasma membrane	238	1564	4.25E-34

Supplementary Table 4. Top 10 KEGG enrichment results of differentially expressed genes.

Term ID	Description	Observed gene count	Background gene count	FDR
hsa04060	Cytokine-cytokine receptor interaction	87	263	8.54E-30
hsa04640	Hematopoietic cell lineage	45	94	3.85E-20
hsa04062	Chemokine signaling pathway	58	181	2.48E-19
hsa04514	Cell adhesion molecules (CAMs)	48	139	4.34E-17
hsa05150	Staphylococcus aureus infection	32	51	4.34E-17
hsa05323	Rheumatoid arthritis	38	84	1.04E-16
hsa04672	Intestinal immune network for IgA production	28	44	3.6E-15
hsa05332	Graft-versus-host disease	25	36	3.21E-14
hsa05140	Leishmaniasis	31	70	1.47E-13
hsa05330	Allograft rejection	23	35	8.55E-13

Supplementary Table 5. Top 10 enriched GO terms in PPI network in the category molecular function of differentially expressed genes.

Term ID	Description	Observed gene count	Background gene count	FDR
GO:0038023	signaling receptor activity	46	1429	7.63E-14
GO:0004888	transmembrane signaling receptor activity	37	1226	2.30E-10
GO:0004896	cytokine receptor activity	13	91	2.30E-10
GO:0042287	MHC protein binding	7	31	1.27E-06
GO:0003823	antigen binding	8	56	2.29E-06
GO:0005102	signaling receptor binding	34	1513	2.29E-06
GO:0019956	chemokine binding	6	22	3.32E-06
GO:0015026	coreceptor activity	7	42	4.47E-06
GO:0004950	chemokine receptor activity	6	27	7.06E-06
GO:0019955	cytokine binding	9	99	7.06E-06

Supplementary Table 6. Top 10 enriched GO terms in PPI network in the category biological process of differentially expressed genes.

Term ID	Description	Observed gene count	Background gene count	FDR
GO:0002376	immune system process	90	2370	5.10E-38
GO:0006955	immune response	73	1560	1.34E-34
GO:0002682	regulation of immune system process	65	1391	5.24E-30
GO:0046649	lymphocyte activation	39	358	7.23E-29
GO:0050776	regulation of immune response	51	873	8.34E-27
GO:0002250	adaptive immune response	34	280	2.22E-26
GO:0002684	positive regulation of immune system process	49	882	8.66E-25
GO:0006952	defense response	55	1234	7.00E-24
GO:0045321	leukocyte activation	48	894	1.09E-23
GO:0001775	cell activation	50	1024	4.19E-23

Supplementary Table 7. Top 10 enriched GO terms in PPI network in the category cellular component of differentially expressed genes.

Term ID	Description	Observed gene count	Background gene count	FDR
GO:0071944	cell periphery	101	5254	2.62E-20
GO:0005886	plasma membrane	99	5159	7.33E-20
GO:0044459	plasma membrane part	69	2651	1.67E-18
GO:0044425	membrane part	107	6517	2.66E-17
GO:0009897	external side of plasma membrane	22	223	2.07E-15
GO:0098552	side of membrane	28	436	2.21E-15
GO:0031224	intrinsic component of membrane	91	5316	1.86E-14
GO:0016021	integral component of membrane	88	5155	1.09E-13
GO:0031226	intrinsic component of plasma membrane	47	1641	2.24E-13
GO:0016020	membrane	115	8420	2.89E-13

Supplementary Table 8. Top 10 KEGG results in PPI network of differentially expressed genes.

Term ID	Description	Observed gene count	Background gene count	FDR
hsa05340	Primary immunodeficiency	10	37	3.78E-10
hsa04060	Cytokine-cytokine receptor interaction	17	263	5.16E-09
hsa04659	Th17 cell differentiation	12	102	5.16E-09
hsa04660	T cell receptor signaling pathway	12	99	5.16E-09
hsa04658	Th1 and Th2 cell differentiation	11	88	9.65E-09
hsa04640	Hematopoietic cell lineage	11	94	1.53E-08
hsa04514	Cell adhesion molecules (CAMs)	12	139	5.34E-08
hsa04612	Antigen processing and presentation	8	66	2.03E-06
hsa05321	Inflammatory bowel disease (IBD)	7	62	1.76E-05
hsa04650	Natural killer cell mediated cytotoxicity	8	124	0.00013

Supplementary Table 9. Literature supports five genes associated with the prognosis of hepatocellular carcinoma.

Gene Symbol	Publication
GZMA	Cytolytic Activity (CYT) Score Is a Prognostic Biomarker Reflecting Host Immune Status in Hepatocellular Carcinoma (HCC).
CD79A	Hepatosplenic Gamma Delta T-Cell Lymphoma (HSGDTCL): Two Rare Case Reports from Western India.
IGJ	Prediction of Response to Sorafenib in Hepatocellular Carcinoma: A Putative Marker Panel by Multiple Reaction Monitoring-Mass Spectrometry (MRM-MS).
CYP3A4	CYP3A4 Gene Is a Novel Biomarker for Predicting a Poor Prognosis in Hepatocellular Carcinoma.
SPP1	A four-gene-based prognostic model predicts overall survival in patients with hepatocellular carcinoma.
