# Supplementary material for: TCGA and ESTIMATE data mining to identify potential prognostic biomarkers in HCC patients
Source: Aging (Albany NY). 2020 Nov 11;12(21):21544–58. doi: 10.18632/aging.103943 (PMC7695391; doi:10.18632/aging.103943)
Supplement: Supplementary File 1 [file aging-12-103943-s002..docx]

**Supplementary File 1. 214 DEGs and their P value.**

| gene | pvalue |
| --- | --- |
| CD4 | 2.80E-05 |
| ITM2A | 2.80E-05 |
| PTGER2 | 0.000104 |
| S1PR4 | 0.000224 |
| CFHR3 | 0.000258 |
| XCR1 | 0.000299 |
| MLC1 | 0.000334 |
| PADI2 | 0.000372 |
| CD226 | 0.000421 |
| BEND4 | 0.000433 |
| OLFM1 | 0.000454 |
| CD3G | 0.00071 |
| PRF1 | 0.000719 |
| FLT3 | 0.000846 |
| C16orf54 | 0.000904 |
| GIMAP1 | 0.001072 |
| TBX21 | 0.001232 |
| SLAMF6 | 0.001345 |
| PRR33 | 0.001433 |
| TRAF3IP3 | 0.001435 |
| CRHBP | 0.001472 |
| FCRL6 | 0.001507 |
| ACKR1 | 0.001649 |
| CCR2 | 0.001748 |
| SH2D1A | 0.001805 |
| C11orf21 | 0.00185 |
| SIDT1 | 0.001908 |
| BLK | 0.002051 |
| IL18RAP | 0.002157 |
| PRKCQ | 0.002326 |
| IL16 | 0.002386 |
| ICAM3 | 0.002447 |
| CARD11 | 0.002482 |
| NLRC3 | 0.002483 |
| CD48 | 0.002592 |
| TRAT1 | 0.002598 |
| CD5L | 0.002658 |
| PI16 | 0.002736 |
| BMP5 | 0.003051 |
| UBASH3A | 0.003163 |
| P2RX1 | 0.003192 |
| PYHIN1 | 0.003295 |
| GPR18 | 0.003325 |
| IGJ | 0.003404 |
| MAP4K1 | 0.003573 |
| CXCR3 | 0.003635 |
| CLEC9A | 0.003674 |
| NKG7 | 0.003675 |
| FAM65B | 0.003887 |
| CD3E | 0.004164 |
| SPN | 0.004315 |
| GLT6D1 | 0.004554 |
| GFRA2 | 0.004635 |
| SELP | 0.004696 |
| CCR5 | 0.00492 |
| IL7R | 0.005109 |
| IL12RB1 | 0.005137 |
| PDZRN3 | 0.005177 |
| CD5 | 0.005964 |
| CD79A | 0.006098 |
| CAMK4 | 0.006193 |
| IGLL5 | 0.006239 |
| GZMH | 0.006256 |
| CLEC12A | 0.006381 |
| KLRK1 | 0.006414 |
| IPCEF1 | 0.006534 |
| FCN3 | 0.006592 |
| GPR171 | 0.006762 |
| OTOG | 0.006875 |
| BTLA | 0.007246 |
| NTRK2 | 0.007321 |
| ACAP1 | 0.007326 |
| ZAP70 | 0.007548 |
| HGFAC | 0.00784 |
| NRROS | 0.008204 |
| LY9 | 0.008485 |
| OGN | 0.008583 |
| ZNF831 | 0.008753 |
| KLRD1 | 0.008795 |
| LILRB5 | 0.008892 |
| TESPA1 | 0.00913 |
| PCDH7 | 0.009333 |
| PHACTR3 | 0.009503 |
| FCER1A | 0.009601 |
| ZBP1 | 0.009688 |
| INMT | 0.011068 |
| ATP2A3 | 0.011272 |
| SAMD3 | 0.012379 |
| LCK | 0.012566 |
| CCBE1 | 0.012734 |
| SVEP1 | 0.0129 |
| KLRB1 | 0.013573 |
| C15orf48 | 0.014231 |
| MAL | 0.014473 |
| PLD4 | 0.014772 |
| OMG | 0.015119 |
| PDZD4 | 0.015254 |
| HTRA4 | 0.015796 |
| GZMB | 0.015966 |
| RYR1 | 0.016007 |
| C9 | 0.016012 |
| LGR6 | 0.016026 |
| FCRL1 | 0.016259 |
| IL10RA | 0.016489 |
| FGL2 | 0.016795 |
| RASAL3 | 0.016828 |
| MS4A1 | 0.016935 |
| GABRP | 0.017052 |
| SLAMF1 | 0.017527 |
| CYP3A4 | 0.018149 |
| TDO2 | 0.018224 |
| NCKAP1L | 0.018436 |
| RCAN2 | 0.018461 |
| EOMES | 0.018576 |
| HCLS1 | 0.018952 |
| PMAIP1 | 0.019186 |
| CD96 | 0.019333 |
| LILRA4 | 0.019391 |
| ATP1A3 | 0.019815 |
| ASB2 | 0.020099 |
| GZMA | 0.020258 |
| GZMK | 0.020369 |
| RTN1 | 0.020542 |
| CST7 | 0.020556 |
| CIITA | 0.021052 |
| IFI44L | 0.021142 |
| SPIC | 0.021542 |
| TNFRSF17 | 0.022015 |
| PHLDA2 | 0.022198 |
| APBA2 | 0.022932 |
| CFP | 0.023224 |
| KRT27 | 0.023342 |
| BIN2 | 0.023405 |
| ARHGAP15 | 0.024578 |
| APOBEC3H | 0.024735 |
| TCL1A | 0.025122 |
| CD6 | 0.025266 |
| LMNTD1 | 0.025581 |
| CXCR6 | 0.025952 |
| SDS | 0.026087 |
| PDCD1LG2 | 0.026279 |
| P2RY10 | 0.026495 |
| NME8 | 0.026854 |
| SLC1A5 | 0.027107 |
| TSPAN32 | 0.027681 |
| KCNA3 | 0.028426 |
| WBSCR17 | 0.028521 |
| TIMD4 | 0.028613 |
| TTC16 | 0.028969 |
| ITK | 0.02915 |
| FUT6 | 0.029247 |
| CLEC4E | 0.029383 |
| PDZRN4 | 0.029732 |
| SIT1 | 0.0298 |
| SIGLEC8 | 0.03009 |
| CD74 | 0.030131 |
| MS4A7 | 0.030444 |
| RUNX2 | 0.030573 |
| WDFY4 | 0.030965 |
| SHISA3 | 0.031106 |
| CYTIP | 0.03118 |
| DCN | 0.031505 |
| CD8B | 0.031981 |
| WAS | 0.032142 |
| C11orf96 | 0.032337 |
| PTGDR | 0.032728 |
| KIAA1755 | 0.032939 |
| SLAMF7 | 0.033022 |
| LCN2 | 0.033758 |
| MPEG1 | 0.0339 |
| SAMD9L | 0.033955 |
| PLCB2 | 0.033999 |
| CD69 | 0.034263 |
| TMIGD2 | 0.034856 |
| DAPP1 | 0.035016 |
| LAX1 | 0.035079 |
| IL21R | 0.035328 |
| GPR174 | 0.035937 |
| CXorf65 | 0.036706 |
| CLEC10A | 0.036893 |
| ABI3BP | 0.038773 |
| CARD17 | 0.039287 |
| RHOH | 0.039775 |
| OMD | 0.040157 |
| LCP1 | 0.040232 |
| CCR7 | 0.040575 |
| CD247 | 0.040915 |
| CEACAM4 | 0.041405 |
| RGS18 | 0.042329 |
| ST6GALNAC1 | 0.042583 |
| THEMIS | 0.042605 |
| PRKAR2B | 0.042742 |
| KIAA1644 | 0.042987 |
| PIK3R5 | 0.043428 |
| TIFAB | 0.043831 |
| TAGAP | 0.044052 |
| C7 | 0.044205 |
| HLA-DOA | 0.044656 |
| FAM129C | 0.044761 |
| CD70 | 0.045525 |
| FYB | 0.045926 |
| RSPO2 | 0.046016 |
| FCN1 | 0.046097 |
| CD40LG | 0.046277 |
| FCRLA | 0.046613 |
| RTP5 | 0.047101 |
| RNF43 | 0.047538 |
| HLA-DPA1 | 0.04785 |
| SPP1 | 0.048235 |
| CD8A | 0.04922 |
| FAM78A | 0.049542 |
| TNFSF8 | 0.049647 |
| B3GALT2 | 0.049952 |
| IL18 | 0.049982 |
