# Supplementary material for: TCGA and ESTIMATE data mining to identify potential prognostic biomarkers in HCC patients
Source: Aging (Albany NY). 2020 Nov 11;12(21):21544–58. doi: 10.18632/aging.103943 (PMC7695391; doi:10.18632/aging.103943)
Supplement: Supplementary File 2 [file aging-12-103943-s003..pdf]

SUPPLEMENTARY FILE

Supplementary File 2. The 8 genes primer sequence were as follows.

CD3E-F	TGCTGCTGGTTTACTACTGGA
CD3E-R	GGATGGGCTCATAGTCTGGG
KLRD1-F	CAGGACCCAACATAGAACTCCA
KLRD1-R	GGAAATGAAGTAACAGTTGCACC
PRKCQ-F	ATGTCGCCATTTCTTCGGATT
PRKCQ-R	ACATACTCTTTGACGAGCACAG
ITK-F	GAAGATCGTCATGGGAAGAAGC
ITK-R	CGGGTATTTATAGTGGCATGGG
TNFSF8 -F	CACGAGCCGCAGCTATTTCTA
TNFSF8 -R	CTCTGAACGACCAACACCATAA
TRAF3IP3-F	ACCGTGGTACTCAGACAAAGG
TRAF3IP3-R	GCAACTCCGTAATTGGTTTGCT
PHLDA2-F	ACGACATGAAATCCCCCGAC
PHLDA2-R	CCCGCGCTTCTTCTTCCATA
C11orf96-F	CTGCTCCAGTTACCAGGCG
C11orf96-R	TGCAGGAACGACTTCTTGGC
